# Supplementary material for: Heat adaptation of phage T7 under an extended genetic code
Source: Virus Evol. 2021 Dec 1;7(2):veab100. doi: 10.1093/ve/veab100 (PMC8923235; doi:10.1093/ve/veab100)
Supplement: veab100_Supp [file veab100_supp.zip › Sequencing_Analysis.zip!/Sequencing_Analysis/A1/evidence/JC_SIDE_1_52.html]

BRESEQ :: Evidence


| Predicted mutation | | | | | | |
| --- | --- | --- | --- | --- | --- | --- |
| evidence | seq id | position | mutation | annotation | gene | description |
| MC JC | NC\_001604.1 | 1,257 | Δ1,480 bp | intergenic (–/–) | – / – | –/– |

| Missing coverage evidence... | | | | | | | | | | |
| --- | --- | --- | --- | --- | --- | --- | --- | --- | --- | --- |
|  |  |  | seq id | start | end | size | ←reads | reads→ | gene | description |
| \* | \* | ÷ | NC\_001604.1 | 1257 | 2736 | 1480 | 3465 [0] | [3] 3461 | *–/–* | –/– |

| New junction evidence | | | | | | | | | | | |
| --- | --- | --- | --- | --- | --- | --- | --- | --- | --- | --- | --- |
|  | | seq id | position | reads (cov) | reads (cov) | score | skew | freq | annotation | gene | product |
| \* | ? | NC\_001604.1 | = 1256 | 0 (0.000) | 3355 (1.140) | 393/574 | 0.0 | 100% | intergenic (–/–) | *–/–* | –/– |
|  | ? | NC\_001604.1 | 2737 = | 0 (0.000) |  |  |  |  | intergenic (–/–) | *–/–* | –/– |

Only 100 of 3465 total aligned reads displayed.  

`TTTTCGACCACGCTTACGAAATGCTGAAAGAAAACATCCGTTATGATGACATCCGTGACACTGATGACCTGCACGATGCTATTCACATGGCTGCCGATAATGCAGTTCCGCACTACTACGCTGACATCTTTAGCGTAATGGCAAGTGAGGGCATTGACCTTGAGTTCGAAGACTCTGGTCTGATGCCTGACACCAAGGACGTAATCCGCATCCTGCAAGCGCGTATCTATGAGCAATTAACGATTGACCTCTGGGAAGACGCAGAAGACTTGCTCAATGAATACTTGGAGGAAGTCGAGGAGTACGAGGAGGATGAAGAGTAATGTCTACTACCAACGTGCAATACGGTCTGACCGCTCAAACTGTACTTTTCTATAGCGACATGGTGCGCTGTGGCTTTAACTGGTCACTCGCAATGGCACAGCTCAAAGAACTGTACGAAAACAACAAGGCAATAGCTTTAGAATCTGCTGAGTGATAGACTCAAGGTCGCTCCTAGCGAGTGGCCTTTATGATTATCACTTTACTTATGAGGGAGTAATGTATATGCTTACTATCGGTCTACTCACCGCTCTAGGTCTAGCTGTAGGTGCA  >  NC_001604.1/956‑1549`  

                                                                                                                                                                                                                                                                                                            `|`                                                                                                                                                                                                                                                                                                       
                  `aaaTGCTAAAAGAAAACATCCGTTATGATGACATAAGTGAAACTGATGACCTGCACGATGCTATTCACATGGCTGCCGATAATGCAGTTCCGCACTACTACGCTGACATCTTTAGCGTAATGGCAAGTGAGGGCATTGACCTTGAGTTCGAAGACTCTGGTCTGCTGCCTGACACCAAGGACGTAATCCACATCCTGCAAGCGCGTATCTATGAGCAATTAACGATTGACCTCTGGGAAGACGCAGAAGACTTGCTCAATGAATACTTGGAGGAAGTCGAGGAagtcgcacgacagaaaga                                                                                                                                                                                                                                                                                     <  1:133301‑M1/301‑19 (MQ=255)`  
                       `cTGAAAGAAAACAACCGGTATGATTACAGCGGTGGCAGTGATGAGCTGCACGATGCTATTCACATGGCTGCCGATAATGCAGTACCGCACGACTAAGCTGACGTCGGTAGCGTAAGAGCAAGTGAGGGGATTGACCTTGAATTCGAAGACTCTGGTCTGATGCGTGACACCAAAGACGTAATCCGGATCCTGCAAGCGCGTATCTATGAGCAATTAAAGATTGACCTCTGGGAAGACGCAGAAGACTTGCTCAATGAATACTTGGAAAAAGTCGAGAAagtcgcacgacagaaagaaattg                                                                                                                                                                                                                                                                                <  1:409299‑M1/301‑24 (MQ=255)`  
                         `gaaagaaaACATACGTTATGATGACATCCGTGACACTGATGACCTGCACGATGCTATTCACATGGCTGCCGATAATGCAGTTCCGCACTACTACGCTGACATCTTTAGCGTAATGGCAAGTGAGGGCATTGACCTTGAGTTCGAAGACTCTGGTCTGATGCCTGACACCAAGGACGTAATCCGCATCCTGCAAGCGCGTATCTATGAGCAATTAACGATTGACCTCTGGGAAGACGCAGAAGACTTGCTCAATGAATACTTGGAGGAAGTCGAGGAagtcgcacgacagaaagaaattgac                                                                                                                                                                                                                                                                              <  1:476633‑M1/301‑26 (MQ=255)`  
                           `gctcggagaggtgtataagagacagccgtcacactgatgaccTGCACGATGCTATTCACATGGCTGCCGATAATGTAGTTCCGCACTACTACGCTGACATCTTTAGCGTAATGGCAAGTGAGGGCATTGACCTTGAGTTCGAAGACTCTGGTCTGATGCCTGACACCAAGGACGTAATCCGCATCCTGCAAGCGCGTATCTATGAGCAATTAACGATTGACCTCTGGGAAGACGCAGAAGACTTGCTCAATGAATACTTGGAGGAAGTCGAGGAagtcgcacgacagaaagaaattgaccg                                                                                                                                                                                                                                                                            <  1:214932‑M1/281‑28 (MQ=255)`  
                                    `tgtgtataagagacagccgtgacaCTGATGACCTGCACGATGCTATTCACATGGCTGCCGATAATGCAGTTCCGAACTACTACGCTGACATCTTTAGCGTAATGGCAAGTGAGGGCATTGACCGTGAGTTCGAAGACTCTGGTCTGATGCCTGACACCAAGGACGTAATCCGCATCCTGCAAGCGCGTATCTATGAGCAATTAACGATTGACCTCTGGGAAGACGCAGAAGACTTGCTCAATGAATACTTGGAGGAAGTAGAGGAagtcgcacgacagaaagaaattgaccgcgctaaggc                                                                                                                                                                                                                                                                   <  1:362266‑M1/290‑37 (MQ=255)`  
                                       `gTTATGATGACATCCGTGACACTGATGACCTGCACGATGCTATTCACATGGCTGCCGATAATGCAGTTCCGCACTACTACGCTGACATCTTTAGCGTAATGGCAAGTGAGGGCATTGACCTTGAGTTCGAAGACTCTGGTCTGATGCCTGACACCAAGGACGTAATCCGCATCCTGCAAGCGCGTATCTATGAGCAATTAACGATTGACCTCTGGGAAGACGCAGAAGACTTGCTCAATGAATACTTGGAGGAAGTCGAGGAagtcgcacgacagaaagaaattgaccgcgctaaggcccg                                                                                                                                                                                                                                                                >  1:281275‑M1/1‑262 (MQ=255)`  
                                            `gattaCATCCGTGACACTGATGACCTGCACGATGCTATTCACATGGCTGCCGATAATGCAGTTCCGCACTACTACGCTGACATCTTTAGCGTAATGGCAAGTGACGGCATTGACCTTGAGTTCGAAGACTCTGGTCTGTTGCCTGACACCAAGGCCGTATTCCGCATCCTGCAAGCGCTTATCTATGAGCAATTAACGAATGACATCTGGGAAGACGCAGAAGACTTGCTCCATGAATCCTTGGAGGAAGTCGATGAagtcgcacgacagaaacaaattgatcccgctaaggcccgcaaaa                                                                                                                                                                                                                                                           >  1:259135‑M1/1‑257 (MQ=255)`  
                                                        `ctcggagatgtgtataagagacagattcacatggctgccgataatgcagTTCCGCACTACTACGCTGACATCTTTAGCGTAATGGCAAGTGAGGGCATTGACCTTGAGTTCGAAGACTCTGGTCTGATGCCTGACACCAAGGACGTAATCCGCATCCTGCAAGCGCGTATCTATGAGCAATTAACGATTGACCTCTGGGAAGACGCAGAAGACTTGCTCAATGAATACTTGGAGGAAGTCGAGGAagtcgcacgacagaaagaaattgaccgcgctaaggcccgtaaagaacgtcacgagg                                                                                                                                                                                                                                               <  1:189294‑M1/277‑57 (MQ=255)`  
                                                          `cacgaatgaccggaAGGATGCTATTCAAATGGCTGCAGATAATGCAGTTCCGAACTACTACGATGACATCTTTAGCGTAATGGCAAGTGAGGGCATTGACCTTGAGTTAGAAGACTCTAGTCTGATGCCTGACACCAAGGACGTAATCCGCATCATGCAAGCGCGTATCTATGAGCAAGTAACGATTGACCTCTGGGAAGACGCAGAAGACTTGCTCAATGAATACTTGGAGGAAGTCGAGGAagtcgaacgacagaaagaaattgaccgcgctaaggcccgtaaagaacgtcacgagggg                                                                                                                                                                                                                                             <  1:137262‑M1/296‑59 (MQ=255)`  
                                                            `cTGATGACCTGCACGATGCTATTCACATGGCTGCCGATAATGCAGTTCCGCACTACTACGCTGACATCTTTAGCGTAATGGCAAGTGAGGGCATTGACCTTGAGTTCGAAGACTCTGGTCTGATGCCTTACACCAAGGACGTAATCCGCATCCTGCAAGCGCGTATCTATGAGCAATTAACGATTGACCTCTGGGAAGACGCAGAAGACTTGCTCAATGAATACTTGGAGGAAGTCGAGGAagtcgaacgacagaaagaaattgaccgcgctaaggcccgtaaagaacgtcacgaggggcg                                                                                                                                                                                                                                           >  1:433300‑M1/1‑241 (MQ=255)`  
                                                            `cTGATGACCTGCACGATGCTATTCACATGGCTGCCGATAATGCAGTTCCGCACTACTACGCTGACATCTTTAGCGTAATGGCAAGTGAGGGCATTGACCTTGAGTTCGAAGACTCTGGTCTGATGCCTGACACCAAGGACGTAATCCGCATCCTGCAAGCGCGTATCTATGAGCAATTAACGATTGACCTCTGGGAAGACGCAGAAGACTTGCTCAATGAATACTTGGAGGCAGTAGAGGAagtcgcacgacagcaagaaatagacggcgccaaggcccgtaaagctgtctcttatacaca                                                                                                                                                                                                                                           >  1:452458‑M1/1‑241 (MQ=255)`  
                                                                      `gCACGATGCTATTCACATGGCTGCCGATAATGCAGTTCCGCACTACTACGCTGACATCTTTAGCGTAATGGCAAGTGAGGGCATTGACCTTGAGTTCGAAGACTCTGGTCTGATGCCTGACACCAAGGACGTAATCCGCATCCTGCAAGCGCGTATCTATGAGCAATTAACGATTGACCTCTGGGAAGACGCAGAAGACTTGCTCAATGAATACTTGGAGGAAGTCGAGGAagtcgcacgacagaaagaaattgaccgcgctaaggcccgtaaagaacgtaacgaggggcgcttagaggcg                                                                                                                                                                                                                                 >  1:278723‑M1/1‑231 (MQ=255)`  
                                                                      `gCACGATGCTATTCACATGGCTGCCGATAATGCAGTTCCGCACTACTACGCTGACATCTTTAGCGTAATGGCAAGTGAGGGCATTGACCTTGAGTTCGAAGACTCTGGTCTGATGCCTGACACCAAGGACGTAATCCGCATCCTGCAAGCGCGTATCTATGAGCAATTAACGATTGACCTCTGGGAAGACGCAGAAGACTTGCTCAATGAATACTTGGAGGAAGTCGAGGAagtcgcacgacagaaagaaattgacagcgctaaggcccgtaaagaacgtcacgcggggcggttagaggcc                                                                                                                                                                                                                                 >  1:85333‑M1/1‑231 (MQ=255)`  
                                                                             `gCTATTCACATGGCTGCCGATAATGCAGTTCCGCACTACTACGCTGACATCTTTAGCGTAATGGCAAGTGAGGGCATTGACCTTGAGTTCGAAGACTCTGGTCTGATGCCTGACACCAGGGACGTAATCCGCATCCTGCAAGCGCGTATCTATGAGCAATTAACGATTGACCTCTGGGAAGACGCAGAAGACTTGCTCAATGAATACTTGGAGGAAGTCGAGGAagtcgcacgacagaaagaaattgaccgcgctaaggcccgtaaagaacgtcacgagctgtcccttatccccatctccc                                                                                                                                                                                                                          >  1:305319‑M1/1‑224 (MQ=255)`  
                                                                             `gCTATTCACATGGCTGCCGATAATGCAGTTCCGCACTACTACGCTGACATCTTTAGCGTAATGGCAAGTGAGGGCATTGACCTTGAGTTCGAAGACTCTGGTCTGATGCCTGACACCAAGGACGTAATCCGCATCCTGCAAGCGCGTATCTATGAGCAATTAACGATTGACCTCTGGGAAGACGCAGAAGACTTGCTCAATGAATACTTGGAGGAAGTCGAGGAagtcgcacgacagaaagaaattgaccgcgctaaggcccgtaaagaacgtcacgaggggcgcttagaggcacgcagat                                                                                                                                                                                                                          >  1:500449‑M1/1‑224 (MQ=255)`  
                                                                                `aTTCACATGTCTGCCGATAATGCAGTTCCGCACTACTACGCTGACATCTTTAGCGTAATGGCAAGTGAGGGCATTGACCTTGAGTTCGAAGACTCTGGTCTGATGCCTGACACCAAGGACGTAATCCGCATCCTGCAAGCGCGTATCTATGAGCAATTAACGATTGACCTCTGGGAAGACGCAGAAGACTTGCTCAATGAATACTTGGAGGAAGTCGAGGAagtcgcacgacagaaagaaattgaccgcgctaaggcccgtaaagaacgttccgaggggcgcttagaggcacgcagatcca                                                                                                                                                                                                                       >  1:5318‑M1/1‑221 (MQ=255)`  
                                                                                   `cacaTGGATGCCGATAATGCAGTGCCGAACTACTACGCTGACATCTTTAGCGTAATGGCAAGTGAGGGAATTGACCTTGGGTTCGAAGACTCTGGTCTGATGACTGACACCAAGGACGTAATCCGCATACTGAAAGGGCGTATCGATGGGCAAGTAACGGTTGACCTCTGGGAAGACGCAGAAGACTTGCTCAATGAATACTTAGAGGAAGTCGAGGAagtcgcaagacagaaagaaattgaccgcgctaaggcccgtaaagaacgtcacgaggggcgcttagaggcacgcagattcaaac                                                                                                                                                                                                                    <  1:70290‑M1/301‑84 (MQ=255)`  
                                                                                              `cGATAATGCAGTTCCGCACTACTACGCTGACATCTTTAGCGTAATGGCAAGTGAGGGCATTGACCTTGAGTTCGAAGACTCTGGTCTGATGCCTGACACCAAGGACGTAATCCGCATCCTGCAAGCGCGTATCTATGAGCAATTAACGATTGACCTCTGGGAAGACGTAGAAGACTTGCGCAATGAATACTTGGAGGAAGTCGAGGAagtcgcacgatagaaagaaattaaccgcgctaaggaccgtaaagaacgtcacgagggagcttagaggcacgccgattcaaacgtcgcaaccgaa                                                                                                                                                                                                         >  1:503826‑M1/1‑207 (MQ=255)`  
                                                                                               `gATAATGCAGTTCCGCACTACTACGCTGACATCTTTAGCGTAATGGCAAGTGAGGGCATTGTCCTTGAGTTCGAAGCCTCTGGTCTGATGCCTGACACCAAGGACGTAATCCGCATCCTGCAAGCGCGTATCTATGAGCAATTAACGATTGACCTCTGGGAAGACGCAGAAGACTTGCTCAATGAATACTTGGAGGAAGTCGAGGAagtcgcacgacagaaagaaattgaccgcgctaaggcccgtaaagaacgtcacgaggggcgattagaggcacgcagattcaaacgtcgcaaccgca                                                                                                                                                                                                        >  1:142652‑M1/1‑206 (MQ=255)`  
                                                                                                         `gatacagctactacgctgacATCCTTAGCGTAATGGCAAGTTAGGGCATTGACCTTGAGTTCGAATACCCTGGTCTGATTCCAGACACCAAGGACGTAATCCGCATCCTGCAAGCGCGTATCTATGAGCAATTAACGATTGACCTCTGGGAAGACGCAGAAGACTTGCTCAATGAATACTTGGAGGAAGTCGAGGAagtcgcacgacagaaagaaattgaccgcgctaaggcccgtaaagaacgtcacgaggggcgcttagaggtacgcagattcaaacgtcgcaaccgcaaggcacgtaa                                                                                                                                                                                              <  1:44711‑M1/294‑106 (MQ=255)`  
                                                                                                          `tCCGCACTACTACGCTGACATCTTTAGCGTAATGGCAAGTGAGGGCATTGACCTTGAGTTCGAAGACTCTGGTCTGATGCCTGACACCAAGGACGTAATCCGCATCCTGCAAGCGCGTATCTATGAGCAATTAACGATTGACCTCTGGGAAGACGCAGAAGACTTGCTCAATGAATACTTGGAGGAAGTCGAGGAagtcgcacgacagaaagaaattgaccgcgctaaggcccgtaaagaacgtcacgaggggcgcttagaggcacgcagattcaaacgtcgcaaccgcaaggcacgtaaa                                                                                                                                                                                             >  1:458205‑M1/1‑195 (MQ=255)`  
                                                                                                           `gagatgtgtagaagagacagctttagcgtaaGGGCAAGTGAGGGCATTGACCTTGAGTTCGAAGACTCGGGGCTGATGCCTGACACCAAGGACGTAATCCGCATCCTGCAAGCGAGTATCAATGAGCAAGTAACGATTGACCTCTGGGAAGACGCAGAAGACTTGCTCAAGGAATACTTGGAGGAAGTCGAGGAagtcgcacgacagaaagaaattgaccgcgctaaggcccgtaaagaacgtcacgaggggcgcttagaggcactcagattcaaacgtcgcaaccgcaaggcacgtaaag                                                                                                                                                                                            <  1:209873‑M1/286‑108 (MQ=255)`  
                                                                                                           `cctcactACTACGCTGACATCTTTAGCGTAATGGCAAGTGAGGGCATTGACCTTGAGTTCGAAGACTCTGGTCTGATGCCTGACACCAAGGACGTAATCCGCATCCTGCAAGCGCGTATCTATGAGCAACTAACGATTGACCTCTGGGAAGACGCAGAAGACTTGCTCAATGAATACTTGGAGGAAGTCGAGGAagtcgcacgacagaaagaaattgaccgcgctaaggcccgtaaagaacgtcacgaggggcgcttagaggcacgcagattccaacgtcgcaaccgcaaggcacgtaaag                                                                                                                                                                                            >  1:239329‑M1/4‑194 (MQ=255)`  
                                                                                                           `ccGCACTACTACGCTGACATCTTTAGCGTAATGGCAAGTGAGGGCATTTACCTTGAGTTCGAAGACTCTGGTCTGATGCCTGACACCAAGGACGTAATCCGCATCCTGCAAGCGCGTATCTATGAGCAATTAACGATTGACCTCTGGGAAGACGCAGAAGACTTGCTCAATGAATACTTGGAGGAAGTCGAGGAagttgcacgacagaaagaaattgaccgcgctaaggcccgtaaagaacgtcacgaggggcgcttagaggcacgcagattcaaacgtcgcaaccgaaaggcacgtaaaa                                                                                                                                                                                            >  1:344351‑M1/1‑194 (MQ=255)`  
                                                                                                              `cACTACTACGCTTACATCTTTAGCGGAATGGAAAGGGAGGGCATTGACATTGAATTCGAAGACTCTGGTCTGATGCCTGACACCAAGGACGTAATCCGCATCCTGCAAGCGCGTATCTATGAGCTATTAACGATTGACCTCTGGGAAGACGCAGAAGACTTGCTCAATGAATACTTGGAGGAAGTAGAGGAagtcgcacgacagaaagaaattgaccgcgctaaggcccgtaaagaacgtcacgaggggcgcttagaggcacgcagattcaaacgtcgcaaccgcaaggcacgtaaagcac                                                                                                                                                                                         <  1:312979‑M1/301‑111 (MQ=255)`  
                                                                                                                `ctactaCGCTGACATCTTTAGCGTAATGGCAAGTGAGGGCATTGACCTTGAGTTCGAAGACTCTGGTCTGATGCCTGACACCAAGGACGTAATCCGCATCCTGCAAGCGCGTATCTATGAGCAATTAACGATTGACCTCTGGGAAGACGCAGAAGACTTGCTCAATGAATACTTGGAGGAAGTCGAGGAagtagcacgacagaaagaaattgaccgcgctaaggcccgtaaagaacgtcacgaggggcgcttagaggcacgcagattcaaacgtcgcaaccgcaaggcacgtaaagcacac                                                                                                                                                                                       >  1:301341‑M1/1‑189 (MQ=255)`  
                                                                                                                  `actccGCTGACATCTTTAGCGTAATGGCAAGTGAGGGCATTGACCTTGAGTTCGAAGACTCTGGTCTGATGCCTGACACCAAGGATGTAATCCGCATCCTGCAAGCGCGTATCTATGAGCAATTAACGATTGACCTCTGGGAAGACGCAGAAGACTTGCTCAATGAATACTTGGAGGAAGTCGAGGAagtcgcacgacagaaagtaattgaccgcgctaaggcccgtaaagaacgtcacgaggggcgctaggaggcacgaagagtcaaccgtgccaccccaaaggcacggaaagaccaaaa                                                                                                                                                                                     >  1:105270‑M1/1‑187 (MQ=255)`  
                                                                                                                       `gCTGACATCTTTAGCGTAATGGCAAGTGAGGGCATTGACCTTGAGTTCGAAGACTCTGGTATGATGCCTGACACCAAGGACGTAATCCGCATCCTGCAAGCGCGTATCTATGAGCAATTAACGATTGACCTCTGGGAAGACGCAGAAGACTTGCTCAATGAATACTTGGAGGAAGTCGAGGAagtcgcacgacagaaagaaattgaccgcgctaaggcccgtaaagaacgtcacgaggggcgcttagaggcacgcagattcaaacgtcgcaaccgcaaggcacgtaaagcacacaaagcta                                                                                                                                                                                <  1:32109‑M1/301‑120 (MQ=255)`  
                                                                                                                          `gACATCTTTAGCGTAATGGCAAGTGAGGGCATTGACCTTGAGTTCGAAGACTCTGGTCTGATGCCTGACACCAAGGACGTAATCCGCATCCTGCAAGCGCGTATCTATGAGCAATTAACGATTGACCTCTGGGAAGACGCAGAAGACTTGCTCAATGAATACTTGGAGGAAGTCGAGGAagtcgcacgacagaaagaaattgaccgcgctaaggcccgtaaagaacgtcacgaggggcgcttagaggcacgcagattcaaacgtcgcaaccgcaaggcaagtaaagcacaaaaagctaagg                                                                                                                                                                             >  1:466350‑M1/1‑179 (MQ=255)`  
                                                                                                                              `tCTTTAGCGTAATGGCAAGTGAGGGCATTGACCTTGAGTTCGAAGACTCTGGTCTGATGCCTGACACCAAGGACGTAATCCGCATCCTGCAAGCGCGTATCTATGAGCAATTAACGATTGACCTCTGGGAAGACGCAGAAGACTTGCTCAATGAATACTTGGAGGAAGTCGAGGAagtcgcacgacagaaagaaattgaccgagctacggcccgtaaagaacgtcacgaggggcgcttagaggcacgcagattcaaacgtcgcaaccgcaaggcacgtaaagcacacaaagctaagcgctg                                                                                                                                                                         >  1:485423‑M1/1‑175 (MQ=255)`  
                                                                                                                               `cTTTAGCGTAATGGCAAGTGAGGGCATTGACCTTGAGTTCGAAGACTCTGGTCTGATGCCTGACACCAAGGACGTAAACCGCATCCTGCAAGCGCGTATCTATGAGCAATTAACGATTGACCTCTGGGAAGACGCAGAAGACTTGCTCAATGAATACTTGGAGGAAGTCGAGGAagtcgcacgacagaaagaaattgaccgcgctaaggcccgtaaagaacgtcacgaggggcgcttagaggcacgcagattcaaacgtcgcaaccgcaaagcacgtaaagcacacaaagctaagcgcgaa                                                                                                                                                                        >  1:297788‑M1/1‑174 (MQ=255)`  
                                                                                                                                 `ttAGCGTAATGGCAAGTGAGGGCATTGACCTTGAGTTCGAAGACTCTGGTCTGATGCCTGACACCAAGGACGTAATCCGCATCCTGCAAGCGCGTATCTATGAGCAATTAACGATTGACCTCTGGGAAGACGCAGAAGACTTGCTCAATGAATACTTGGAGGAAGTCGAGGAagtcgcacgacagaaagaaattgaccgcgctaaggcccgtaaagaacgtcacgagtggcgcttagaggcacgcagattcaaacgtcgcaaccgcaaggcacgtaaaggacacaaagctaagcgcgaaaa                                                                                                                                                                      >  1:255054‑M1/1‑172 (MQ=255)`  
                                                                                                                                    `gCGTAATGGCAAGTGAGGGCATTGACCTTGAGTTCGAAGACTCTGGTCTGATGCCTGACACAAAGGACGTAATCCGCATCCTGCAAGCGCGTATCTATGAGCAATTAACGATTGACCTCTGGGAAGACGCAGAAGACTTGCTCAATGAATACTTGGAGGAAGTCGAGGAagtcgcacgacagaaagaaattgaccgcgctaaggcccgtaaagaacgtcacgaggggcgcttagaggcacgcagattcaaacgtcgcaaccgcaaggaacgtaaagcacacaaagctaagcgcgaaagaaa                                                                                                                                                                   >  1:443086‑M1/1‑169 (MQ=255)`  
                                                                                                                                          `tGGCAAGTGAGGGCATTGACCTTGAGTTTGAAGACTCTGGTCTGATGCCTGACACCAAGGACGTAATCCGCATCCTGCAAGCGCGTATCTATGAGCAATTTACGATTGACCTCTGGGAAGACGCAGAAGACTTGCTCAATGAATACTTGGAGGAAGTAGTGGAagtcgcacgacagaatgaaattgaccgcgctaaggccggtaaagaacgttatgaggggcgcttagaggcacgcagcttcaaacgtcgtaaacgcaaggaaggtaaagcacacaaagctaagcgtgaaaacatgcttgc                                                                                                                                                             >  1:427847‑M1/1‑163 (MQ=255)`  
                                                                                                                                          `tGGAAAGCGATGGCATTGACCTTGAGTTCGAAGACTCTGGTCTGATGCCTGAAACCAAGGACGTAATCCGCACACGTCAAGCGCGTATCTATGAGCAATTAACGATTGACCGCTGGGAAGACGCAGAAGACTTGCTCAATGAAAACTTGGAGGAAGTCGAGGAagtcgcacgacagaaagaaattgaccgcgctaaggcccgtaaagaacgtcacgaggggcgcttagaagcacgcagattcaaacgtcgcaaccgcaaggcacgtaaagcacacaaagctaagcgcgaaagaatgcttgc                                                                                                                                                             <  1:40078‑M1/301‑139 (MQ=255)`  
                                                                                                                                                     `ggCATTGACCTTGAGTTCGAAGACTCTGGTCTGATGCCTGACACCAAGGACGTAATCCGCATCCTGCAAGCGCGTATCTATGAGCAATTAACGATTGACCTCTGGGAAGACGCAGAAGACTTGCTCAATGAATACTTGGAGGAAGTCGAGGAagtcgcacgacagaaagaaattgaccgcgctaaggcccgtaaagaacgtcacgcggggcgcttagaggcacgcagattcaaacgtcgcaaccgcaaggcacgtaaagcacacaaagctaagcgctaaagaatgctttctgcgtggcgac                                                                                                                                                  >  1:129095‑M1/1‑152 (MQ=255)`  
                                                                                                                                                     `ggCATTGACCTTGAGTTCGAAGACTCTGGTCTGATGCCTGACACCAAGGACGTAATCCGCATCCTGCAAGCGCGTATCTATGAGCAATTAACGATTGACCTCTGGGAAGACGCAGAAGACTTGCTCAATGAATACTTGCAGGAAGTCGAGGAagtcgcacgacagaaagaaattgaccgcgctaaggcccgtaaagaacgtcacgaggggcgcttagaggcacgcagattcaaacgtcgcaaccgcaaggcacgtaaagcacacaaagctaagcgcgaaagaatgcttgctgcgctgtctc                                                                                                                                                  >  1:422838‑M1/1‑152 (MQ=255)`  
                                                                                                                                                           `gACCTTGAGTTCGAAGACTCTGGTCTGATGCCTGACACCAAGGACGTAATCCGCATCCTGCAAGCGCGTATCTATGAGCAATTAACGATTGACCTCTGGGAAGACGCAGAAGACTTGCTCAATGAATACTTGGAGGAAGTCGATGAagtcgcacgacagaaagaaattgaccgcgctaaggcccgtaaagaacgtcacgaggggcgcttagaggcacgcagattcaaacgtcgcaaccgcaaggcacgtacagcacacaaagctaagcgcgaaagaatgcttgctgcgtggcgatgggctg                                                                                                                                            >  1:308186‑M1/1‑146 (MQ=255)`  
                                                                                                                                                                     `tCGAAGACTCTGGTCTGATGACTGACACCAAGGACGTAATCCGCATCCTGCAAGCGCGTATCTATGAGCAATTAACGATTGACCTCTGGGAAGACGCAGAAGACTTGCTCAATGAATACTTGGAGGAAGTCGAGGAagtcgcacgacagaaagaaattgaccgcgctaaggcccgtaaagaacgtcacgatgggcgcttagaggcacgcagattcaaacgtcgcaaccgcaaggcacgtaaagcacacaaagataagcgcgaaagaatgcttgctgcgtggcgatgggctgaacgtcaaga                                                                                                                                  <  1:404828‑M1/301‑166 (MQ=255)`  
                                                                                                                                                                       `gAAGACTCTGGTCTGATGCCTGACACCAAGAACTTAATCAGCATCCTGCAAGCGCGTATCTATGAGCAATTACCGATTGCCCTCAGGGAAGACCCAGAAGACTTGCTAAAGGAATACTTGGAGGAAGTCGAGGAagtcgcacgacagaaagaaattaaccgcgctaaggccagtaaagaacgtcacgaggggcgcttagaggcacgcagattcaaacgtcgcaaccgaaaggcacgtaaagcacacaaagctaagagcgaaagaatgcttgctgcgtggcgatgggctgaacgtcaagaac                                                                                                                                <  1:65927‑M1/301‑168 (MQ=255)`  
                                                                                                                                                                           `aCTCTGGTCTGATGCCTGACACCAAGGACGTAATCCGCATCCTGCAAGCGCGTATCTATGAGCAATTAACGATTGACCTCTGGGAAGACGCAGAAGACTTGCTCAATGAATACTTGGAGGAAGTCGAGGAagtcgcacgacagaaagaaattgaccgcgctaaggcccgtaaagaacgtcacgaggggcgcttagaggcacgcagattcaaacgtcgcaaccgcaaggcacgtaaagcacacaaagctaagcgcgaaagaatgcttgctgcgtggcgatgggctgaacgtcaagaacggcg                                                                                                                            >  1:418031‑M1/1‑130 (MQ=255)`  
                                                                                                                                                                               `gggtCTGATGCCTGACACCAAGGACGTAATCCGCATCCTGAAAGCGCGTATCTATGAGCAATTAACGATTGACCTCTGGGAAGACGCAGAAGACTTGCTCAATGAATACTTGGAGGAAGTCGAGGAagtcgcacgacagaaagaaattgaccgcgctaaggcccgtaaagaacgtcacgaggggcgcttagaggcacgcagattcaaacgtcgcaaccgcaaggcacgtaaagcacacaaagctaagcgcgaaagaatgcttgctgcgtggcgatgggctgaacgtcaagaacggcgtaac                                                                                                                        <  1:183384‑M1/300‑176 (MQ=255)`  
                                                                                                                                                                                `ggtgtgatccccaacccaaaggccgtaatccgcgtcctAGAAGCGCGTAGCCATAGACAATTAACGGTTGACCTCTGGGAAGACGCAGAAGACTTGCTCAATGAATACTTGAGGGAAGTCGAGGAagtcgcacgacagaaagaaattgaccgcgctaaggcccgtaaagaacgtcacgaggggcgcttagaggcacgcagattcaaacgtcgcaaccgcaaggcacgtaaagcacacaaagctaagcgcgaaagaatgcttgctgcgtggcgatgggctgaacgtcaagaacggcgtaacc                                                                                                                       <  1:267040‑M1/283‑177 (MQ=255)`  
                                                                                                                                                                                `ggTCTGATGCCTGACACCAAGGACGTAATCCGCATCCTGCAAGCGCGTATCTATGAGCAATTAACGATTGACCTCTGGGAAGACGCAGAAGACTTGCTCAATGAATACTTGGAGGAAGTCGAGGAagtcgcacgacagaaagaaattgaccgcgctaaggcccgtaaagaacgtcacgaggggcgcttagaggcacgcagattcaaacgtcgcaaccgcaaggcacgtaaagcacacaaagctaagcgcgaaagaatgcttgctgcgtggcgatgggctgaacgtcaagaaaggcgtaacc                                                                                                                       >  1:56414‑M1/1‑125 (MQ=255)`  
                                                                                                                                                                                     `gATGCCTGACACCAAGGACGTAATCCGCATCCTGCAAGCGCGTATCTATGAGCAATTAACGATTGACCTCTGGGAAGACGCAGAAGACTTGCTCAATGAATACTTGGAGGAAGTCGAGGAagtcgcacgacagaaagaaattgacagcgctaaggcccgtaaagaacgtcacgaggggcgcttagaggcacgcagattcaaacgtcgcaaccgcaaggcacgtaaagcacacaaagctaagagcgaaagaatgcttgctgcgtggcgatgggctgaacgtcaagaacggcgtaaccactgt                                                                                                                  >  1:508876‑M1/1‑120 (MQ=255)`  
                                                                                                                                                                                      `ttgCATGACACCAAGGACGTAATCCGAAGACTGCAAGCGAGTATCTATGAACAATTAACGATTGACCTCTGGGAAGACGAAGAAGACTTGCAAAATGAATACTTGGAGGAAGTCGAGGAagtcgcacgacagaaagaaattgaccgcgctaaggcccgtaaagatcgtcacgagtggcgcttagaggcacgcagattcatacgtcgcaaccgcaaggcacgtaaagcacacaaagctaagcgcgaaagaatgcttgctgcgtggcgatgggctgaacgtcaagaacggcgtaaccatgagg                                                                                                                 <  1:75764‑M1/300‑183 (MQ=255)`  
                                                                                                                                                                                      `aTGCCTGACACCAAGGACGTAATCCGCATCCTGCAAGCGCGTATCTATGAGCACTTAACGATTGACCTCTGGGAAGACGAAGAAGACTTGCTCAATGAATACTTGGAGGAAGTCGCGGAagtcgcacgacagacagaaattgaccgcgataaggcccgtaaagaacggcccgaggggcgattagaggcacgcagattcaaacgtcgcaaccgcaaggcacgaaaagcacacaaagctaagcgcgaaagaatgcttgctgcgtggcgatgggctgaacgtcaagaacggcgtaacctgtctc                                                                                                                 >  1:385857‑M1/1‑119 (MQ=255)`  
                                                                                                                                                                                      `aTGCCTGACACCAAGGACGTAATCCGCATCCTGCAAGCGCGTATCTATGAGCAATTAACGATTGACCTCTGGGAAGACGCAGAAGACTTGCTCAATGAATACTTGGAGGAAGTCGAGGAagtcgcacgacagaaagaaattgaccgcgctaaggcccgtaaagaacgtcacgaggggcgcttagaggcacgcagattcaaacgtcgcaaccgcaaggcacgtaaagcacacaaagctaagcgcgaaagaatgcttgctgcgtggcgatgggctgaacatcaagaacggcgtaaccatgagg                                                                                                                 <  1:390770‑M1/301‑183 (MQ=255)`  
                                                                                                                                                                                      `aTGCCTGACACCAAGGACGTAATCCGCATACTGCAAGCGCGTATCTATGAGCAATTAACGATTGACCGCTGGGTAGACGCAGAAGACTTTCTCAATGAATACTTGGAGGAAGTCGAGGAattagcacgacagaaagaaattggccgcgctaaggcccgtaaagaacgtcacgaggggcgcttagaggcacgcagattcaaacgtcgcaaccgcaaggcacgtaaagcacacaaagctaagcgcgaaagaatgcttgctgcgtggcgatgggctgaacgtcaagaacggcgtaaccatgagg                                                                                                                 <  1:290374‑M1/301‑183 (MQ=255)`  
                                                                                                                                                                                       `agcCTGACACCAAGGACTTAACCCGCATCCTGCAAGCGCGTATCTATGAGCAATTAACGATTGACCGCTGGGAAGACGCAGAAGACTTGCTCAATGAATACTTGGAGGAAGTCGAGGAagtcgcacgacagaaagaaattgaccgcgctaaggcccgtaaagaacgtcacgaggggcgcttagaggcacgcagattcaaacgtcgcaaccgcaaggcacgtaaagcacacaaagctaagcgcgaaagaatgcttgctgcgtggcgatgggctgaacgtcaagaacggcgtaaccatgaggt                                                                                                                <  1:317812‑M1/300‑184 (MQ=255)`  
                                                                                                                                                                                       `agcCTGACACCAAGGACGTAATCCGCATCCTGCAAGCGCGTATCTATGAGCAATTAACGATTGACCTCTGGGAAGACGCAGAAGACTTGCTCAATGAATACTTGGAGGAAGTCGAGGAagtcgcacgacagaaagaaattgaccgcgctaaggcccgtaaagaacgtcacgaggggcgcttagaggcacgcagattcaaacgtcgcaaccgcaaggcacgtaaagcacacaaagctaagcgcgaaagaatgcttgctgcgtggcgatgggctgaacgtcaagaacggcgtaaccatgaggt                                                                                                                <  1:481332‑M1/300‑184 (MQ=255)`  
                                                                                                                                                                                        `gCCTGACACCAAGGACGTAATCCGCATCCTGCAAGCGCGTATCTATGAGCAATTAACGATTGACCTCTGGGAAGACGCAGAAGACTTGCTCAATGAATACTTGGAGGAAATCGAGGAagtcgcacgacagaaagaaattgaccgcgctaaggcacgtaaagaacgtcacgcggggcgcttagaggcacgcagattcaaacgtcgcaaccgcaagtcacgtaaagcacacaaagctaagcgcgaaagaatgcttgctgcgtgtcgatgggcttaacttccagaacggcgtaaccatgaggta                                                                                                               >  1:81820‑M1/1‑117 (MQ=255)`  
                                                                                                                                                                                              `cacCAAGGACGTAATCCGCATCCTGCAAGCGCGTATCTATGAGCAATTAACGATTGACCTCTGGGAAGACGCAGAAGACTTGCTCAATGAATACTTGGAGGAAGTCGAGGAagtcgcacgacagaaagaaattgaccgcgctaaggcccgtaaagaacgtcacgaggggcgcttagaggcacgcagattcaaacgtcgcaaccgcaaggcacgtaaagcacacaaagctaagcgcgaaagaatgcttgctgcgtggcgatgggctgaacgtcaagaacggcgtaaccatgaggtagctggt                                                                                                         >  1:225108‑M1/1‑111 (MQ=255)`  
                                                                                                                                                                                              `cacCAAGGACGTAATCCGCATCCTGCAAGCGCGTATCTATGAGCAATTAACGATTGACCTCTGGGAAGACGCAGAAGACTTGCTCAATGAATACTTGGAGGAAGTCGAGGAagtcgcacgacagaaagaaattgaccgcgctaaggcccgtaaagaacgtcacgaggggcgcttagaggcacgcagattcaaacgtcgcaaccgcaaggcacgtaaagcacacaaagctaagcgcgaaagaatgcttgctgcgtggcgatgggctgaacgtcaagaacggaataacctgtttcttatacac                                                                                                         >  1:166849‑M1/1‑111 (MQ=255)`  
                                                                                                                                                                                                 `cAAGGACGTAATCCGCATCCTGCAAGCGCGTATCTATGAGCAATTAACGATTGACCTATGGGAAGACGCAGAAGACTTGCTCAATGAATACTTGGAGGAAGTCGAGGAagtcgcacgacagaaagaaagtgaccgcgctaaggcccgtaaagaacgtcacgaggggcgcttagaggcacgcagattcaaacgtcgcaaccgaaaggcacgtaaagcacacaaagctaagcgcgaaagaatgcttgctgcgtggcgatgggctgaacgtcaagaacgacgtaaccatgaggtagctgtagat                                                                                                      <  1:471941‑M1/301‑194 (MQ=255)`  
                                                                                                                                                                                                 `aaagGACATAATCCGCATCCTGCAAGCGCGTATCTATGAGCAATTAACGATTGACCTCTGGGAAGACGCAGAAGACTTGCTCAATGAATACTTGGAGGAAGTCGAGGAagtcgcacgacagaaagaaattgaccgcgctaaggcccgtaaagaacgtcacgaggggcgcttagaggcacgcagattcaaacgtcgcaaccgcaaggcacgtaaagcacacaaagctaagcgcgaaataatgcttgctgcgtggcgatgggctgaacgtcaagaacggcgtaaccatgaggtagctgtagat                                                                                                      <  1:324841‑M1/300‑194 (MQ=255)`  
                                                                                                                                                                                                  `aaGGACGTAATCCCCATCCTGCAAGCGCGTATCTATGAGAAATTAACGATTGACCTATGGGAAGACGAAGAAGACTTGCTCAATGAATACTTGGAGGAAGTCGAGGAagtcgcaggacagaaagaaattgaccgcgctaaggcccgtaaagaacgtcacgaggggcgcttagaggaacgcagattcaaacgtcgcaaccgcaaggcacgtaaagcacacaaagctaagcgcgaaagaatgcttgctgcgtggcgatgggctgaacgtcaagaacggcgtaaccatgaggtagctgtagatg                                                                                                     <  1:205093‑M1/301‑195 (MQ=255)`  
                                                                                                                                                                                                  `aaGGACGTAAGCCGCAGCCGGCAAGCACTAAACTATGAGCAATTAACGATTGACCTCTGGGAAGACGCAGAAGACTTGCTCAATGAATACTTGGAGGAAGTCGAGGAagtcgcacgacagaaagaaattgaccgcgctaaggcccgtaaagaacgtcacgaggggcgcttagaggcacgcagattcaaacgtcgcaaccgcaaggcacgtaaagcacacaaagctaagcgcgaaagaatgcttgctgcgtggcgatgggctgaacgtcaagaacggcgtaaccatgaggtagctgtagatg                                                                                                     <  1:329759‑M1/301‑195 (MQ=255)`  
                                                                                                                                                                                                    `ggACGTAATCCGCATCCTGCAAGCGCGTATCTATGAGCAATTAACGATTGACCTCTGGGAAGACGCAGAAGACTTGCTCAATGAATACTTGGAGGAAGTCTAGGAagtcgcacgacagaaagaaattgaccgcgctaaggcccgtaaagaacgtcacgaggggcgcttagaggcacgcagattcaaacgtcgcaaccgcaaggcacgtaaagcacacaaagctaagcgcgaaagaatgcttgctgcgtggcgatgggctgaacgtcaagaacggcgtaaccatgaggtagctgtagatgta                                                                                                   >  1:367796‑M1/1‑105 (MQ=255)`  
                                                                                                                                                                                                    `ggACGTAATCCGCATCCTGCAAGCGCGTATCTATGAGCAATTAACGATTGACCTCTGGGAAGACGCAGAAGACTTGCTCAATGAATACTTGGAGGAAGTCGAGGAagtcgcacgacagaaagaaattgaccgcgctaaggcccgtaaagaacgtcacgaggggcgcttagaggcacgcagattcaaacgtcgcaaccgcaaggcacgtaaagcacacaaagctaagcgcgaaagaatgcttgctgcgtggcgatgggctgaacgtcaagaacggcgtaaccactgtctcttatacccatcc                                                                                                   >  1:413846‑M1/1‑105 (MQ=255)`  
                                                                                                                                                                                                     `gACGTAATCCGCATCCTGCAAGCGCGTATCTATGAGCAATTAACGATTGACCTCTGGGAAGACGCAGAAGACTTGCTCAATGAATACTTGGAGGAAGTCGAGGAagtcgcacgacagaaagaaattgaccgcgctaaggcccgtaaagaacgtcacgaggggcgcttagaggcacgcagattcaaacgtcgcaaccgcaaggcacgtaaagcacacaaagctaagcgcgaaagaatgcttgctgcgtggcgatgggctgaacgtcaagaacggcgtaaccatgaggtagctgtagatgtac                                                                                                  >  1:338330‑M1/1‑104 (MQ=255)`  
                                                                                                                                                                                                       `cGTAATCCGCATCCTGCAAGCGCGTATCTATGAGCAATTAACGATTGACCTCTGGGAAGACGCAGAAGACTTGCTCAATGAATACTTGGAGGAAGTCGAGGAagtcgaacgacagaaagaaattgaccgcgctaaggcccgtaaagaacgtcacgaggggcgcttagaggcacgcagattcaaacgtcgcaaccgcaaggcacgtaaagcacacaaagctaagctcgaaagaatgcttgctgcgtggcgatgggctgaacgtcaagaacggcgtaaccatgaggtagctgtagatgtacta                                                                                                <  1:100754‑M1/301‑200 (MQ=255)`  
                                                                                                                                                                                                          `aaTCCGCTTCCTGCAAGCGCTTCTCTATTAGCAATTAACGATTGACCTCTGGGAAGACGCAGAAGACTTGCTCAATGAATACTTGGAGGAAGTCGATGAagtcgcacgacagaaagaaattgaccgcgctaaggcccgtaaagaacgtcacgaggggcgcttagaggcacgcagattcaaacgtctcaaccgcaaggcacgtaaagcacacaaagctaagcgcgaaagaatgcttgctgcgtggcgatgggctgaacctcaagaacggcgtaaccatgaggtagctgtagatgtactagga                                                                                             <  1:115108‑M1/301‑203 (MQ=255)`  
                                                                                                                                                                                                          `aaTCCGCATCCTGAAAGCGCGTATCTATGAGAAATTAACGATTGACCTCTGGGAAGACGCAGAAGACTTGCTCAATGAATACTTGGAGGAAGTCGAGGAagtcgcacgacagaaagaaattgaccgagctaaggcccgtaaagaacgtcacgaggggagcttagaggcacgcagattcaaacgtcgcaaccgcaaggcacgtaaagcacacaaagctaagcgcgaaagaatgcttgctgcgtggcgatgggctgaacgtcaagaacggcgtaaccatgaggtagctgtagatgtactagga                                                                                             <  1:153383‑M1/301‑203 (MQ=255)`  
                                                                                                                                                                                                            `tCCGCATCCTGCAAGCGCGTATCTATGAGCAATTAACGATTGACCTCTGGGAAGACGCAGAAGACTTGCTCAATGAATACTTGGAGGAAGTCGAGGAagtcgcacgacagaaagaaattgaccgcgctaaggcccgtaaagaacgtcacgaggggcgcttagaggcacgcagattcaaacgtcgcaaccgcaaggcacgtaaagcacacaaagctaagcgcgaaagaatgcttgctgcgtggcgatgggctgaacgtcaagaacggcgtaaccatgaggtagctgtagatgtactaggaag                                                                                           >  1:438116‑M1/1‑97 (MQ=255)`  
                                                                                                                                                                                                            `tCCGCATCCTGCAAGCGCGTATCTATGAGCAATTAACGATTGACATCTGGGAAGACGCAGAAGACTTGCTCAATGAATACTTGGAGGAAGTCGAGGAagtcgcacgacagaaagaaattgaccgcgctaaggcccgtaaagaacgtcccgaggggcgcttagaggcacgaagattcaaacgtcgcaaccgcaaggcacgtaaagcacacaaagctaagcgcgaaagaatgcttgctgcgtggcgatgggctgaccgtcaagaacggcgtcaccattaggctttcccttatacacctctccc                                                                                           >  1:294227‑M1/1‑97 (MQ=255)`  
                                                                                                                                                                                                                    `cTGAAAACGGGTATATATGATCAATTAAAGAATGACCTATGGGAAGACGAAGAAGACTTGCTCAATGAATACTTGTATGAAGTCTAGGAagtcgcacgacagaaagaaattgaccgcgctaaggcccgtaaagaacgtcacgaggggcgcttagaggcacgcagattcaaacgtcgcaaccgcaatgcacgtaaagcacacaaagctaagcgcgaaagaatgcttgctgcgtggcgatgggctgaacgtcaagaacggcgtaaccatgaggtagctgtagatgtactaggaagaaccaata                                                                                   <  1:251214‑M1/301‑213 (MQ=255)`  
                                                                                                                                                                                                                        `aagagctgatctattagCAATTAATGATTGACCTCTGGGAAGACGAAGAAAAATGACTCAATGAATAATTGGAGGAAGACGAGAAaggcgcacgacagaaagaaattgaccgcgctaaggcgcgaaaaaaacgtcacgaggggcgcttagaggcacgcagattcaaacggcgcaaccgcaaggcacgtaaagcacacaaagctaagcgcgaaagaatgcttgcttcctggcgatgggctgaacgtcaagaacggcgtaaccatgaggtagctgtagatgtactaggaagaaccaataacgc                                                                               <  1:158359‑M1/293‑217 (MQ=255)`  
                                                                                                                                                                                                                         `aGCGCGTATCTATGAGCAATTAACGATTGACCTCTGGGAAGACGCAGAAGACTTGCTCAATGAATACTTGGAGGAAGTCGAGGAagtcgcactacagaaagaaattgaccgcgctaaggcccgtaaataacgtcacgaggggcgcttagaggcacgcagattcaaacgtcgcaaccgcaaggcacgtaaagcacacaaagctaagcgcgaaagaatgcttgctgcgtggcgatgggctgaacgtcaagaacggcgtaaccattaggtagctgtagatgtactaggaagaaccaataacgct                                                                              >  1:334417‑M1/1‑84 (MQ=255)`  
                                                                                                                                                                                                                          `gcgcGTATCTATGAGCAATTAACGATTGACCTCTGGGAAGACGCAGAAGACTTGCTCAATGAATACTTGGAGGAAGTCGAGGAagtcgcacgacagaaagaaattgaccgcgctaaggcccgtaaagaacgtcacgaggggcgcttagaggcacgcagattcaaacgtcgcaaccgcaaggcacgtaaagcacacaaagctaagcgcgaaagaatgcttgctgcgtggcgatgggctgaacgtcaagaacggcgtaaccatgaggtagctgtagatgtactaggaagaaccaataacgcct                                                                             >  1:492436‑M1/1‑83 (MQ=255)`  
                                                                                                                                                                                                                             `ggtATCTATGAGCAATTAACGATTGACCTCTGGGAAGACGCAGAAGACTTGCTCAATGAATACTTGGAGGAAGTCGAGGAagtcgcacgacagaaagaaattgaccgcgctaaggcccgtaaagaacgtcacgaggggcgcttagaggcacgcagattcaaacgtcgcaaccgcaaggcacgtaaagcacacaaagctaagcgcgaaagaatgcttgctgcgtggcgatgggctgaacgtcaagaacggcgtaaccatgaggtagctgtagatgtactaggaagaaccaataacgctatgc                                                                          <  1:21965‑M1/300‑222 (MQ=255)`  
                                                                                                                                                                                                                               `tATCTATGAGCAATTAACGATTGACCTCTGGGAAGACGCAGAAGACTTGCTCAATGAATACTTGCAGGAAGTCGAGGAagtcgcacgaaagaaagaaattgaccgcgctaaggcccgtaaagaacgtcacgagtggcgcgtagaggcacgcagattcaaacggcgcaaccgcaaggcacgtaaagcacacaaagctaagcgcgaaagaatgcttgctgcgtggcgatgggctgaacgtcaagaacggcgtaaccatgaggtagctgtagatgtactaggaagaaccaataacgctatgctc                                                                        <  1:377691‑M1/301‑224 (MQ=255)`  
                                                                                                                                                                                                                                `gctcggagatgtgtataagagacattctgggaagacgcagaagacgtgctcaaTGAATACTTGGAGGAAGTCGAGGAagtcgcacgacagaaagaaattgaccgcgctaaggcccgtaaagaacgtcacgaggggcgcttagaggcacgcagattcaaacgtctcaaccgcaaggcacgtaaagcacacaaagctaagcgcgaaagaatgcttgctgcgtggcgatgggctgaacgtcaagaacggcgtaaccatgaggtagctgtagatgtactaggaagaaccaataacgctatgctct                                                                       <  1:233294‑M1/276‑225 (MQ=255)`  
                                                                                                                                                                                                                                `ataagagacagattaacgattgaCCTATGGGAAGACGCAGAAGACTTGCTCAATGAATACTTGGAGGAAGTCGAGGAagtcgcacgacagaaagaaattgaccgcgctaaggcccgtaaagaacgtcacgaggggcgcttagaggcacgcagattcaaacgtcgcaaccgcaaggcacgtaaagcacacaaagctaagcgcgaaagaatgcttgctgcgtggcgatgggctgaacgtcaagaacggcgtaaccatgaggtagctgtagatgtactaggaagaaccaataacgctaggctct                                                                       <  1:173001‑M1/290‑225 (MQ=255)`  
                                                                                                                                                                                                                                 `tCTATGAGCAATTAACGATTGACCTCTGGGAAGACGCAGAAGACTTGCTCAATGAATACTTGGAGGAAGTCGAGGAagtcgcacgacagaaagaaattgaccgcgctaaggcccgtaaagaacgtcacgaggggcgcttagaggcacgcagattcaaacgtcgcaaccgcaaggcacgtaaagcacacaaagctaagcgcgaaagaatgcttgctgcgtggcgatgggctgaacgtcaagaacggcgtaaccatgaggtagctgtagatgtactaggaagaaccaataacgctatgctctg                                                                      >  1:94224‑M1/1‑76 (MQ=255)`  
                                                                                                                                                                                                                                 `tCTATGAGCAATTAACGATTGACCTCTGGGAAGACGCAGAAGACTTGCTCAATGAATACTTGGAGGAAGTCGAGGAagtcgcacgacagaaagaaattgaccgcgctaaggcccgtaaagaacgtcacgaggggcgcttagaggcacgcagattcaaacgtcgcaaccgcaaggcacgtaaagcacacaaagctaagcgcgaaagaatgcttgctgcgtggcgatgggctgaacgtcaagaacggcgtaaccatgaggtagctgtagatgtactaggaagaaccaataacgctatgctctg                                                                      >  1:247758‑M1/1‑76 (MQ=255)`  
                                                                                                                                                                                                                                        `gCAATTAACGATTGACCTCTGGGAAGACGCAGAAGACTTGCTCAATGAATACTTGGAGGAAGTCGAGGAagtcgcacgacagaaagaaattgaccgcgctaaggcccgtaaagaacgtcacgcggggcgcttagaggcacgcagattcaaacgtcgcaaccgcaaggcacgtaaagcacacaaagctaagcgcgaaagaatgcttgctgcgtggcgatgggctgaacgtcaagaacggcgtaaccatgaggtagctgtagatgtactaggaagaaccaataacgcctgtctcttatacaca                                                               >  1:200060‑M1/1‑69 (MQ=255)`  
                                                                                                                                                                                                                                         `agattAACGAATGAACTATGGGAAGACGCAGAAGACTTGCTAAATGAATACTTGGAGGAAGTCGAGGAagtcgcacgacaaaaagaaattgaccgcactaaggcccgaaaagaacgtcacgcggggcgcttagaggcacgcagattcaaacgtcgcaaccgcaaggcacgtaaagcacacaaagctaagcgcgaaagaatgcttgctgcgtggcgatgggctgaacgtcaagaacggcgtaaccatgaggtagctgtagatgtactaggaagaaccaataacgctatgctctgggtcaaca                                                              <  1:119468‑M1/299‑234 (MQ=255)`  
                                                                                                                                                                                                                                         `aaatTAACGATTGACCTCTGGGAAGACGCAGAAGACTTGCTCAATGAATACTTGGAGGAAGGCGAGGAagtcgcacgacagaaagaaattgaccgcgctaaggcccgtaaagaacgtcacgaggggcgcttagaggcacgcagattcaaacgtcgaaaccgcaaggcacgtaaagcacacaaagctaagcgcgaaagaatgcttgctgcgtggcgatgggctgaacgtcaagaacggcgtaaccatgaggtagctgtagatgtactaggaagaaccaataacgctatgctctgggtcaaca                                                              <  1:195020‑M1/300‑234 (MQ=255)`  
                                                                                                                                                                                                                                          `aaTTAACGATTGAACGCTGGGAAGACGCAGAAGACTTGCTCAAGGAATAATTGGAGGAAGTCGAGGAaggcgcacgacagaaagaaattgaacgcgataaggcacgtaaagaacgtcacgaggggcgcttagaggcacgcagattcaaacgtcgcaaccgcaaggcacgtaaagcacacaaagctaagcgcgaaagaatgcttgctgcgtggcgatgggctgaacgtcaagaacggcgtaaccatgaggtagctgtagatgtactaggaagaaccaataacgctatgctctgggtcaacat                                                             <  1:356327‑M1/301‑235 (MQ=255)`  
                                                                                                                                                                                                                                              `aaCGATTGACCTCTGGGAAGACGCAGAAGACTTGCTCAATGAATACTTGGAGGAAGTCGAGGAagtcgcacgacagaaagaaattgaccgcgctaaggcccgtaaagaacgtcacgaggggcgcttagaggcacgcagattcaaacgtcgcaaccgcaaggcacgtaaagcacacaaagctaagcgcgaaagaatgcttgctgcgtggcgatgggctgaacgtcaagaacggcgtaaccatgaggtagctgtagatgtactaggaagaaccaataacgctatgctctgggtcaacatgttc                                                         <  1:489097‑M1/301‑239 (MQ=255)`  
                                                                                                                                                                                                                                                `cGATGGACCTCTGGTAAGAAGAAGAAAACTTGCTCAATGAATACTGGGAGGAAGTCGAGGAagtcgaatgacagaaagaaattggccgcgctaaggcccgtaaagaacgtcacgaggggcgcttagaggcacgcagattcaaacgtcgcaaccgcaaggcacgtaaagcacacaaagctaagcgcgaaagaatgcttgctgcgtggcgatgggctgaacgtcaagaacggcgtaaccatgaggtagctgtagatgtactaggaagaaccaataacgctatgctctgggtcaacatgttctc                                                       <  1:350734‑M1/301‑241 (MQ=255)`  
                                                                                                                                                                                                                                                `aagccagcctctgggaAGACGCAGAAGACTTGCTCAATGAATACTTGGAGGAAGTCGAGGAagtcgcacgacagaaagaaattgaccgcgctaaggcccgtaaagaacgtcacgaggggcgcttagaggcacgcagattcaaacgtcgcaaccgcaaggcacgcaaagcacacaaagctaagcgcgaaagaatgcttgctgcgtggcgatgggctgaacgtcaagaacggcgtaaccatgaggtagctgtagatgtactaggaagaaccaataacgctatgctctgggtcaacatgttctc                                                       <  1:242796‑M1/294‑241 (MQ=255)`  
                                                                                                                                                                                                                                                       `ccTCTGGGAAGACGCAGAAGACTTGCTCAATGAATACTTGGAGGAAGTCGAGGAagtcgcacgacagaaataaattgaccgcgctaaggcccgtaaagaacgtcacgaggggcgcttagaggcacgcagattcaaacgtcgcaaccgcaaggcacgtaaagcacacaaagctaagcgcgaaagaatgcttgctgcgtggcgatgggctgaacgtcaagaacggcgtaaccatgaggtatctgtagatgtactaggaagaaccaataacgctatgccctgggtcaacatgttctctggggac                                                >  1:147522‑M1/1‑54 (MQ=255)`  
                                                                                                                                                                                                                                                       `ccTCTGGGAAGACGCAGAAGACTTGCTCAATGAATACTTGGAGGAAGTCGAGGAagtcgcacgacagaaagaaattgaccgcgctaaggcccgtaaggaacgtcacgaggggcgcttagaggcacgcagattcaaacatcgcaaccgcaaggcacgtaaagcacacaaagctaagcgcgaaagaatgcttgctgcgtggcgatgggctgaacgtcaagaacggcgtaaccctgaggtagctgtagatgtactaggacgaaccaataacgctatgccctgggtcaacctgttccctggggac                                                >  1:229993‑M1/1‑54 (MQ=255)`  
                                                                                                                                                                                                                                                        `agatgggAAAACGTAGAAGACTTGATGAATGAATACTTGGAGGAAGTAGAGGAagacgaacgacagaaagaaatcaaccgcggtaaggccagtaaagaacgtcacggggggcgcttagaggcacgaagattcaaacgtcgcaaccgcaaggcacgtaaagcacacaaagctaagcgcgaaagaatgctggctgcgtggagatgggcttaacgtcaagaacggcgtaaccatgaggtagctgtagatgtactaggaagaaccaataacgctatgctctgggtcaacatgttctctggggact                                               <  1:123975‑M1/298‑249 (MQ=255)`  
                                                                                                                                                                                                                                                          `ctGGGAAGACGCAGAAGACTTGCTCAATGAATACTTGCAGGAAGTCGAGGAagtcgcacgacagaaagaaattgaccgcgctaaggcccgtaaagaacgtcacgaggggcgcttagaggcacgcagattcaaacgtcgcaaccgcaaggcacgtaaagcacacaaagctaagcgcgaaagaatgcttgctgcgtggcgatgggctgaacgtcaagaacggcgtaaccatgaggtagctgtagatgtactaggaagaaccaataacgctatgctctgggtcaacatgttctctggggacttt                                             >  1:496331‑M1/1‑51 (MQ=255)`  
                                                                                                                                                                                                                                                              `gAAGACGCAGAAGACTTGCTCAATGAATACTTGGAGGAAGGCGAGGAagtcgcaagacagaaagaaaatgaccgcgataaggcccgtaaagaacgtcacgaggggcgcttagaggcacgcagattcaaacgtcgcaaccgcaaggcacgtaaatcacacaaagctaagcgcgaaagaatgcttgctgcgtggcgatgggctgaacgtcaagaacggcgtaaccatgaggtagctgtagatgtactaggaagaaccaataacgctatgctctgggtcaacatgttctctggggactttaagg                                         <  1:287218‑M1/301‑255 (MQ=255)`  
                                                                                                                                                                                                                                                              `gAAGACGCAGAAGACGGGGTAAAAGAATACGTGGAGGAAGTCGAGGAaggcggacgacagaaagaaagtgaccgcgataaggaccggaaagaacgtcacgaggggcgatgagaggcacgaagattcaaacgtcgaaaccgcaaggcacgtaaagaacacaaagctaagcgctaaagaatgctggctgcgtggcgatgggctgaacgtcaagaacggcgtaaccatgaggtagctgtagatgtactaggaagaaccaagaacgctatgctctgggtcaacatgttctctggggactgtaagg                                         <  1:37310‑M1/301‑255 (MQ=255)`  
                                                                                                                                                                                                                                                              `caggacgCAGAAAAAATGCTCACTGAATACTTGGAGGAAGTCTAGGAagtcgcacgacagaaagaaatggacggcgtgtaggcgcgtgaataacgtcacgaatggaagttagaggcacgcagattcaaacgtcgcaaccgcaaggcacgtaaagcacacacagctaagcgcgaaagaatgctttcttcgtggggatgtgctgaacgtcaagaacggcgtaaccatgaggtagctgtagatgtactaggaagaaccaataacgctatgctctgggtcaacatgttctctggggactttaagg                                         <  1:110422‑M1/298‑255 (MQ=255)`  
                                                                                                                                                                                                                                                                  `aCGCAGAAGACTTGCTCAATGAATACTTGGAGGAAGTCGAGGAagtcgcacgacagaaagaaattgaccgcgctaaggcccgtaaagaacgtcacgaggggcgcttagaggcacgcagattcaaacgtcgcaaccgcaaggcacgtaaagcacacaaagctaagcgcgaaagaatgcttgctgcgtggcgatgggctgaacgtcaagaacggcgtaaccatgaggtagctgtagatgtactaggaagaaccaataacgctatgctctgggtcaacatgttctctggggactttaaggcgct                                     >  1:395505‑M1/1‑43 (MQ=255)`  
                                                                                                                                                                                                                                                                      `aaagagacagcttaatgaaTACTTGGAGGAAGTCGAGGAagtcgcacgacagaaagaaattgaccgcgctaaggcccgtaaagaacgtcacgaggggcgcttagaggcacgcagattcaaacgtcgtaaccgcaaggcacgtaaagcacacaaagctaagcgcgaaagaatgcttgctgcgtggcgatgggctgaacgtcaagaacggcgtaaccatgaggtagctgtagatgtactaggaagaaccaataacgctatgctctgggtcaacatgttctctggggactttaaggcgcttgag                                 <  1:60499‑M1/292‑263 (MQ=255)`  
                                                                                                                                                                                                                                                                        `aagaCTTGCTCAATGAATACTTGGAGGAAGTCGAGGAagtcgcacgacagaaagaaattgaccgctctaaggcccgtaaagaacgtcacgaggggcgcttagaggcacgcagattcaaacgtcgcaaccgcaaggcacgtaaagcacacaaagctaagcgcgaaagaatgcttgctgcgtggcgatgggctgaacgtcaagaacggcgtaaccattaggtagctgtagatgtactaggaagaaccaataacgctatgctctgggtcaacatgttctctggggaatttaaggcgcttgagga                               >  1:462323‑M1/1‑37 (MQ=255)`  
                                                                                                                                                                                                                                                                          `gaCTTGCTCAATGAATACTTGGAGGAAGTCGAGTAagtcgcacgccagaaagaaatttaccgcgctaattcccgtaaagaacgtcacgcggggcgcttagaggcacgcagattcaaacgtcgcaaccgcaaggcacgtaaagcacacaaatctaagcgcgaaagaatgcttgctgcgtgtagattggctgaacgtcaagaacggcgtaaccatgcggtcgctgtagatgtactaggaagaaccaataacgctatgctctgggtccacatgttctctgtggactttaaggctcttgaggaac                             >  1:178404‑M1/1‑35 (MQ=255)`  
                                                                                                                                                                                                                                                                              `tGCTAAATGAATACTTGGAGGAAGTCGAGGAagtcgcacgacagaaagaaattgaccgcgctaaggcccgtaaagaacgtcacgaggggcgcttagaggcacgcagattcaaacgtcgcaaccgcaaggcacgtaaagcacacaaagctaagcgcgaaagaatgcttgctgcgtggcgatgggctgaacgtcaagaacggcgtaaccatgaggtagctgtagatgtactaggaagaaccaataacgctatgctctgggtcaacatgttctctggggactttaaggcgcttgaggaacgaat                         <  1:272955‑M1/301‑271 (MQ=255)`  
                                                                                                                                                                                                                                                                                      `gAATACTTGGAGGAAGTCGAGGAagtcgcacgacagaaagaaattgaccgcgctaaggcccgtaaagaacgtcacgaggggcgcttagaggcacgcagattcaaacgtcgcaaccgcaaggcacgtaaagcacacaaagctaagcgcgaaagaatgcttgctgcgtggcgatgggctgaacgtcaagaacggcgtaaccatgaggtagctgtagatgtactaggaagaaccaataacgctatgctctgggttaacatgttctctggggactttaaggcgcttgaggaacctgtctcttata                 >  1:8935‑M1/1‑23 (MQ=255)`  
                                                                                                                                                                                                                                                                                       `aaTCCTTGGAGGAAGTCGAGGAagtcgcacgacagaaagaaattgaccgcgctaaggcccgtaaagaacgtcacgaggggcgcttagaggcacgcagattcaaacgtcgcaaccgcaaggcacgtaaagcacacaaagctaagcgcgaaagaatgcttgctgcgtggcgatgggctgaacgtcaagaacggcgtaaccatgaggtagctgtagatgtactaggaagaaccaataacgctatgctctgggtcaacatgttctctggggactttaaggcgcttgaggaacgaatcgcgctgca                >  1:162944‑M1/1‑22 (MQ=255)`  
                                                                                                                                                                                                                                                                                             `tGGAGGAAGTCGAGGAagtcgcaagacagaaagaaattgaccgcgctaaggcccggaaagaacgtcacgaggggcgcttagaggaacgcagattcaaacgtcgcaaccgcaaggcacgtaaagcacacaaagctaagcgcgaaagaatgcttgctgcgtggcgatgggctgaacgtcaagaacggcgtaaccatgaggtagctgtagatgtactaggaagaaccaataacgctatgctctgggtcaacatgttctctggggactttaaggcgcttgaggaacgaatcgcgctgcactggcg          <  1:262765‑M1/301‑286 (MQ=255)`  
                                                                                                                                                                                                                                                                                                   `aagtcgaggaagtcgaacgacagaaagaaattgaccgcgctaaggccagtaaagaacgtcccgaggggcgcttagaggcacgcagattcaaacgtcgcaaccgcaaggcacgtaaagcacacaaagctaagcgcgaaagaatgcttgctgcgtggcgatgggctgaacgtcaagaacggcgtaaccatgaggtagctgtagatgtactaggaagaaccaataacgctatgctctgggtcaacatgttctctggggactttaaggcgcttgaggaacgaatcgcgctgcactggcgtaatgc    <  1:381103‑M1/301‑292 (MQ=255)`  
                                                                                                                                                                                                                                                                                                     `gtcgaggaagtcgcacgacagaaagaaattgaccgcgctaaggcccgtaaagaacgtcacgaggggcgcttagaggcacgcagattcaaacgtcgcaaccgcaaggcacgtaaagcacacaaagctaagcgcgaaagaatgcttgctgcgtggcgatgggctgaacgtcaagaacggcgtaaccatgaggtagctgtagatgtactaggaagaaccaataacgctatgctctgggtcaacatgttctctggggactttaaggcgcttgaggaacgaatcgcgctgcactggcgtaatgctg  >  1:284293‑M1/1‑8 (MQ=255)`  

                                                                                                                                                                                                                                                                                                            `|`                                                                                                                                                                                                                                                                                                       
`TTTTCGACCACGCTTACGAAATGCTGAAAGAAAACATCCGTTATGATGACATCCGTGACACTGATGACCTGCACGATGCTATTCACATGGCTGCCGATAATGCAGTTCCGCACTACTACGCTGACATCTTTAGCGTAATGGCAAGTGAGGGCATTGACCTTGAGTTCGAAGACTCTGGTCTGATGCCTGACACCAAGGACGTAATCCGCATCCTGCAAGCGCGTATCTATGAGCAATTAACGATTGACCTCTGGGAAGACGCAGAAGACTTGCTCAATGAATACTTGGAGGAAGTCGAGGAGTACGAGGAGGATGAAGAGTAATGTCTACTACCAACGTGCAATACGGTCTGACCGCTCAAACTGTACTTTTCTATAGCGACATGGTGCGCTGTGGCTTTAACTGGTCACTCGCAATGGCACAGCTCAAAGAACTGTACGAAAACAACAAGGCAATAGCTTTAGAATCTGCTGAGTGATAGACTCAAGGTCGCTCCTAGCGAGTGGCCTTTATGATTATCACTTTACTTATGAGGGAGTAATGTATATGCTTACTATCGGTCTACTCACCGCTCTAGGTCTAGCTGTAGGTGCA  >  NC_001604.1/956‑1549`

  

Alignment Legend

Aligned base mismatch/match (shaded by quality score): `ATCG/ATCG` `< 3 ≤` `ATCG/ATCG` `< 9 ≤` `ATCG/ATCG` `< 17 ≤` `ATCG/ATCG` `< 32 ≤` `ATCG/ATCG` `< 38 ≤` `ATCG/ATCG`

Unaligned base: `atcg`    Masked matching base: `atcg`    Alignment gap: `‑`    Deleted base: `‑`

Reads not counted as support for junction

read\_name Not counted due to insufficient overlap past the breakpoint.

read\_name Not counted due to not crossing MOB target site duplication.
